# Three-dimensional mesenchymal stem cell laden scaffold of icariin sustained-release for bone regeneration

**DOI:** 10.55730/1300-0152.2627

**Published:** 2022-09-02

**Authors:** Yanbing LIU, Yan FANG

**Affiliations:** 1 Department of Spinal Surgery, The Third Hospital of Shijiazhuang, Hebei, China; 2 Department of Cardiac Surgery, The Second Hospital of Hebei Medical University, Hebei, China

**Keywords:** Bone defect, bone marrow mesenchymal stem cells, fibrous scaffold, icariin

## Abstract

**Background/aim:**

Icariin (ICA)-loaded zein/PLGA nanofiber membrane combined with MSCs was prepared by coaxial electrospinning and bioelectrospraying.

**Materials and methods:**

SEM and TEM were used to evaluate the surface morphology and microstructure of the fiber membrane. Ultraviolet spectrophotometry was used to detect drug release. A LIVE/DEAD Viability/Cytotoxicity Kit and fluorescence staining were used to detect cell morphology and activity. Alkaline phosphatase and calcium mineralization deposition were used to evaluate the osteoinductive activity of the scaffold. Dynamic mechanical analysis was used to determine the Young’s modulus, maximum load, and maximum elongation of the prepared scaffold. Western blot was used to detect the related protein expression in MSCs induced by drug-loaded scaffolds.

**Results:**

Good mechanical properties and stability were observed in the prepared drug-loaded scaffolds. SEM showed that there were a considerable number of MSCs dispersed in the scaffold. MSCs were evenly distributed, could grow evenly between fibers, and were arranged orderly along the fibers. Sustained release of ICA confers cell laden scaffold higher ALP activity and ECM mineral deposition through Runx2, OPN and OCN pathways.

**Conclusion:**

Isotropic sustained release of ICA grant cell laden scaffolds rapid bone regeneration compacity which can provide a good osteogenic environment for loaded MSCs.

## 1. Introduction

With the rapid development of orthopedic surgery, bone defect is an urgent problem that needs to be solved. Tumors, trauma, and congenital malformations could lead to bone tissue defects, such as osteoblastoma, developmental dysplasia of the hip, and congenital osteogenesis imperfecta (Prasadh et al., 2018). The “gold standard” for clinical treatment of bone defects is still autologous bone transplantation at present, but this repair method causes secondary damage to patients, and the injury and cost in patients highly increase (Sakkas et al., 2017). The emergence and vigorous development of tissue engineering and regenerative medicine provide a good direction for medical treatment of such defects (Holzapfel et al., 2016).

Nanofibers are the most commonly used tissue engineering materials. Poly (lactic-co-glycolic acid (PLGA) has been approved by the FDA for clinical applications, such as nanodrug delivery system, composite gel scaffolds, and suture materials (Amini-Fazl et al., 2020; Uematsu et al., 2005; Nisak et al., 2018). However, like other biopolymers, PLGA has poor hydrophilicity and no natural cell recognition sites on its surface, resulting in poor cell affinity. Materials with different characteristics could be combined to modify PLGA and improve its surface properties. zein is the main protein extracted from maize germ; it could self-assemble into microspheres, films, fibers, and other structures (Liu et al., 2005). As a natural biomaterial, zein has high biodegradability, low toxicity, and excellent biocompatibility compared with synthetic polymers (Yu et al., 2016). Nanofibers with core-shell structure could be prepared by coaxial electrospinning, which could be used to load and release drugs. The core layer is the hotspot of drug targeting because it allows high drug loading. Therefore, the core-shell structure allows fibers to be used as carriers for drug delivery (Chakraborty et al., 2009). By contrast, the drug loaded in the single-layer fiber is exposed to the surrounding environment on the fiber surface, and the initial burst release of functional molecules is much higher (Beachley et al., 2010).

The effect of biological scaffold depends on its interaction with cells. Static inoculation is one of the most common methods for combining cells with scaffolds (Hao et al., 2017). It has been used to prepare different types of cell scaffolds. However, the technology has some serious deficiencies, such as low survival rate of transplanted cells, uneven cell distribution in three-dimensional structure, and poor cell permeability in each layer (Adebiyi et al., 2011; Loh et al., 2013). Bioelectrospray is a technology that uses live cell suspension to generate liquid droplets containing cells through a charged needle (Lee et al., 2018; Hong et al., 2018). The combination of bioelectrospray and coaxial electrospinning is a promising alternative method to produce drug-loaded scaffolds containing cells. Therefore, the cells and drugs could be evenly distributed between the fibers of the scaffolds, which is conducive to the establishment of an isotropic three-dimensional system and the acceleration of bone tissue repair.

Mesenchymal stem cells (MSCs) play a key role in the initial formation and repair of bones. Endochondral ossification is a mechanism of bone healing. MSCs differentiate into chondrocytes, deposit minerals, promote cartilage calcification, and remodel bone formation (Dimitriou et al., 2005). During intrathecal ossification, MSCs or undifferentiated preosteocytes could directly differentiate into osteoblasts (Thompson et al., 2002). Osteoblasts are formed in the initial stage of bone and play an important role in maintaining bone ossification and fracture repair. They are involved in the repair of persistent microfractures in daily injuries and contribute to the dynamic reconstruction of bones (Bielby et al., 2007). Various bioactive molecules secreted by MSCs also contribute to the creation of the optimal microenvironment for osteogenesis and regeneration (Caplan et al., 2007).

Icariin (ICA) could enhance the proliferation of osteoblasts. Huang et al. (2018) cultured human osteoblasts in vitro and found that ICA could promote the content of alkaline phosphatase (ALP) in the cells, which could provide a good therapeutic direction for bone diseases to a certain extent (Wang et al., 2014). In addition to enhancing cell proliferation, ICA could promote the differentiation of MSCs into osteoblasts and directly accelerate the healing of bone injury. Through the intervention culture of bone marrow MSCs with the main components of *Epimedium* flavonoids, Liang W et al. (Liang et al., 2012) found that the formation of mineralized nodules in the cells could be obviously observed in each treatment group after 14 days. Their result indicated that the main components of flavonoids in *Epimedium* could promote the osteogenic differentiation of MSCs. Network pharmacology analysis showed that ICA could promote the proliferation and differentiation of osteoblasts through five main targets (Yang et al., 2019).

In the present study, a drug-loaded stem cell nanofiber was prepared using coaxial electrospinning technology combined with bioelectrospray technology. Zein/PLGA shell-core structure was used to achieve the controlled release of ICA and promote the osteogenic differentiation of MSCs. The integration of MSCs with drug-loaded electrospun scaffolds (zein-ICA/PLGA-MSC) and the characterization of the prepared scaffolds were analyzed using different methods. The drug release experiment in vitro confirmed that the nanofiber scaffold had a good drug release effect. The biocompatibility of the zein-ICA/PLGA-MSC was detected through cell staining. ALP and alizarin red (ARS) staining were used to qualitatively analyze the osteogenic ability of the prepared nanofiber scaffold and proved that the zein-ICA/PLGA-MSC had good biological activity and osteogenic ability. Western blot was also used to verify the effect of the drug-loaded cell scaffold on the expression of osteogenic protein in MSCs.

## 2. Materials and methods

### 2.1. Preparation of polymer solution

Zein, PLGA, and zein-ICA were dissolved in hexafluoroisopropanol (HFIP). Zein (600 mg) and ICA (2.3 mg) were dissolved in 2.3 mL (3.64 g) of HFIP solution to form zein-ICA solution. A solution containing only zein (0.6 g, 14% wt/wt) and PLGA (0.2 g, 6% wt/wt) was prepared. It was magnetically stirred at room temperature for 4 h. In the experiment, the same mass ratio of shell to core was used in the further preparation. The mass ratio between zein and zein-ICA shell solution and PLGA nuclear solution was 3:1.

### 2.2. Preparation of Zein-ICA/PLGA-MSC scaffold

In the device for coaxial electrospinning, two concentric stainless-steel needles were made into coaxial nozzles. Zein and zein ICA shell solutions were pushed through the external needle at a flow rate of 0.008 mL/min. The PLGA core solution was pushed through the coaxial inner needle at a flow rate of 0.006 mL/min. The nozzle of the device was connected to a high-voltage power supply, and the distance from the needle (inner diameter of 0.6 mm) to the collector plate was 7.5 cm at 15 kV. The following parameters were used to electrospray the MSC suspension (Gibco C57BL/6 (MSC), ThermoFisher, USA): 3.10 mL/h, 4.3 cm, and 16 kV voltage. The cell containing the scaffold was formed and placed on a culture dish on a rotating plate (70 rpm). After 17 min of coaxial electrospinning and cell electrospray, the cell scaffold was submerged using DMEM and cultured in 5% CO_2_ at 37 °C. The scaffolds without cells were prepared only by coaxial electrospinning.

### 2.3. Physicochemical properties of Zein-ICA/PLGA-MSC scaffolds

For physical and chemical analysis, Zein-ICA/PLGA-MSC was washed with phosphate-buffered saline (PBS) immediately after preparation to remove unbounded cells. The samples were then dried at 30 °C for 24 h.

#### 2.3.1. Characterization of Zein-ICA/PLGA-MSC scaffolds

The morphology of the scaffolds was analyzed using scanning electron microscopy (SEM, JEOL JSM-7800F) after spray gold treatment by a sputtering gold plating machine (ISC150). The average diameter of the fibers (n = 30) was measured on ImageJ 1.38 software. The samples were prepared by electrospinning on a 400-mesh carbon-coated copper mesh. The structures of the scaffolds were verified via transmission electron microscopy (TEM, JEM-1400Flash) at 100 kV voltage.

#### 2.3.2. In vitro release of ICA

The samples were cut into 20 mm × 20 mm size, the initial weight was weighed and recorded, and the ICA content of each sample was calculated. The samples were immersed in an EP tube containing 2 mL of PBS and placed in a thermostatic oscillator at 37 °C. The supernatant was collected at a certain interval and then replaced with 2 mL of fresh PBS. Subsequently, 0.5 mL of the collected supernatant was taken out for measurement. The OD value of ICA was determined via UV spectrophotometry (UV-2600i, Shimadzu, Japan) at 270 nm. Four repeated samples were set for each group. The OD value was converted into concentration using standard curve. The results were expressed as cumulative release at each time point.


(1) Cumulative release (%)=MT/Mo×100%,

where MT is the cumulative release of ICA at time point, and Mo is the total amount of ICA contained in each sample. Each sample was measured four times.

#### 2.3.3. In vitro degradation

Zein/PLGA, zein-ICA/PLGA, zein/PLGA-MSC, and zein-ICA/PLGA-MSC were incubated in PBS. At 37 °C, the scaffolds were completely immersed in PBS solution with a pH of 7.4 for degradation in vitro, and the PBS solution was replaced every 48 h. The support in PBS was taken out at different time points, washed with distilled water, and then placed in a vacuum drying oven for dehydration until the weight of the support did not change. The average molecular weight (Mw) of zein/PLGA, zein-ICA/PLGA, zein/PLGA-MSC, and zein-ICA/PLGA-MSC were calculated using gel permeation chromatography (Prominence GPC). The scaffold was dissolved in tetrahydrofuran and eluted at a flow rate of 1 mL/min at 45 °C. Calibration curves were obtained using polystyrene standard.

#### 2.3.4. Mechanical properties

Dynamic mechanical analysis (DMA, n = 5) was used to determine the Young’s modulus, maximum load, and maximum elongation of zein/PLGA, zein-ICA/PLGA, zein/PLGA-MSC, and zein-ICA/PLGA-MSC (Q800AT-DMA). The scaffold was cut into rectangular size (5 × 10 mm). The test was carried out at a slope force of 0.4 N/ min and a static load of 0.005 N until the maximum load of 20 N.

### 2.4. Cell activity in scaffolds

MSC activity was measured by staining the scaffolds with a LIVE/DEAD Viability/Cytotoxicity Kit (Invitrogen, USA). After 7 days of culture, the medium was taken out and washed with PBS three times. The detection reagent was added to the scaffolds. Afterward, the scaffolds implanted with MSC were cultured at 37 °C and 5% CO_2_ for 20 min. The MSCs were observed on an inverted fluorescence microscope (Nikon, Japan).

The MSCs on the scaffolds were washed with PBS and fixed with 4% paraformaldehyde, dried, and observed using SEM. Their morphology at different time points was detected using the AlexaFluor594 method. After 1, 7, and 15 days of culture, the MSCs were washed with PBS and fixed with 4% paraformaldehyde. Afterwards, the scaffolds were soaked in 0.25% Triton X-100 for 20 min, soaked in 1% bovine serum albumin for 40 min, stained with AlexaFluor 594 phalloidin for 1 h and DAPI staining for 5 min, and then observed using an inverted fluorescence microscope.

### 2.5. ALP activity detection

After 15 days of osteogenic induction, the samples were gently washed with PBS and then treated with 0.1% Triton X-100 for 30 min. The cells were blown evenly using a pipette tip, and the liquid was collected in a 1.5-mL centrifuge tube. Centrifugation was carried out in accordance with the instructions from the ALP kit. Subsequently, 50 μL of the supernatant was drawn into a 96-well plate, and 50 mL of PNPP solution was added. After 10–30 min of exposure, the corresponding absorbance was measured at 405 nm using an enzyme-labeled instrument.

### 2.6. Alizarin Red S staining

The calcium nodules were stained with Alizarin Red S(ARS) 15 days after osteogenic induction. The calcium nodules were first fixed with 4% paraformaldehyde at room temperature for 30 min, stained with alizarin red (USA) at 37 °C for 30 min, and then washed with PBS to remove the excess dye. The stained calcium nodules were observed under a microscope. Next, quantitative analysis was carried out, and 10% cetylpyridinium chloride was used to dissolve calcium nodules. The liquid was transferred to a 96-well plate, and the absorbance value was measured at 562 nm via ELISA (Zou et al., 2015).

### 2.7. Western blot analysis

After 15 days of culture, the cells were collected and lysate (1 mL of distilled water + 50 μL of 1 M DTT + 500 μL of 3× lysate) was added to the scaffolds. Ultrasonic fragmentation and preservation at −80 °C were then conducted. The protein concentration of the sample was determined using BCA method, and the sample amount was calculated. Membrane transfer was then conducted via 80 V/30 min + 120 V/1 h of electrophoresis. Afterwards, the residual membrane transfer solution was washed with TBST buffer, and 5% skimmed milk powder was used for sealing at room temperature for 1 h. The first antibody was applied by direct sticking method, and the temperature was 4 °C overnight. A TBST buffer solution was used to wash the membrane three times for 15 min each time. The second antibody was incubated at room temperature for 1 h. The ECL luminescent liquid (Bimake, USA) was developed in the chemiluminescence gel imaging system (Syngene, UK) and analyzed on Image J software.

### 2.8. Statistical analysis

The experimental data were expressed as’x ± S. SPSS 20.0 software was used for statistical analysis. ANOVA was used to compare any difference between groups; *p < 0.05 indicated statistical difference.

## 3. Results

### 3.1. Characterization of Zein-ICA/PLGA-MSC nanofiber scaffold

**Figure 1:**
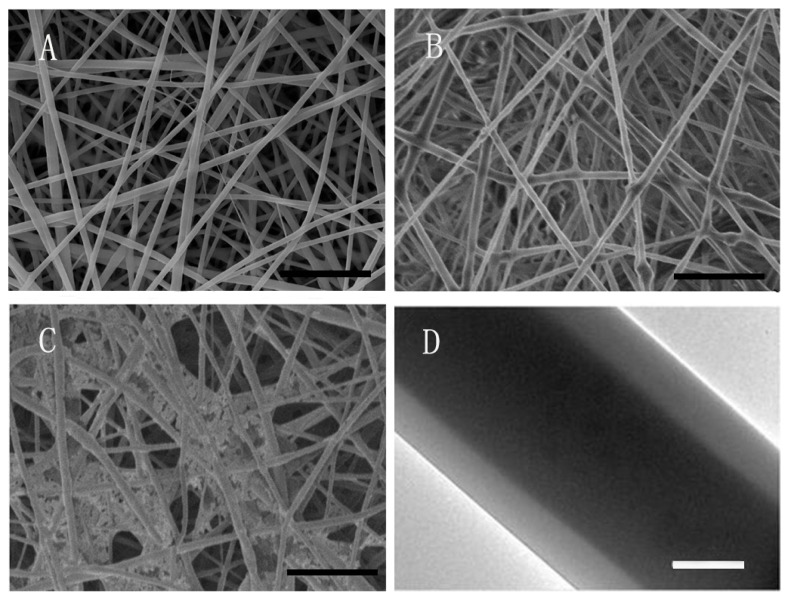
SEM and TEM images of nanofiber scaffold. (A–C) Zein/PLGA, zein-ICA/PLGA, and zein-ICA/PLGA-MSC fiber scaffolds, respectively; bar = 10 μm. (D) TEM images of zein-ICA/PLGA-MSC coaxial nanofiber scaffolds, bar = 500 nm.

The measured values of the zein/PLGA nanofiber scaffold showed the minimum fiber diameter (0.85 ± 0.13 μm), as shown in Figure 1A. The loading of ICA into the material increased the diameter of nanofibers, as shown in Figure 1B. The diameter of the zein-ICA/PLGA nanofibers prepared via coaxial electrospinning was 1.3 ± 0.12 μm. The fiber diameter of the Zein-ICA/PLGA-MSC (1.835 ± 0.16 μm) nanofibers was the highest (Figure 1C). TEM images showed that the Zein-ICA/PLGA-MSC nanofibers fabricated via coaxial electrospinning combined with bioelectrospray technology had a continuous core-shell structure (Figure 1D).

### 3.2. Drug release in vitro

**Figure 2:**
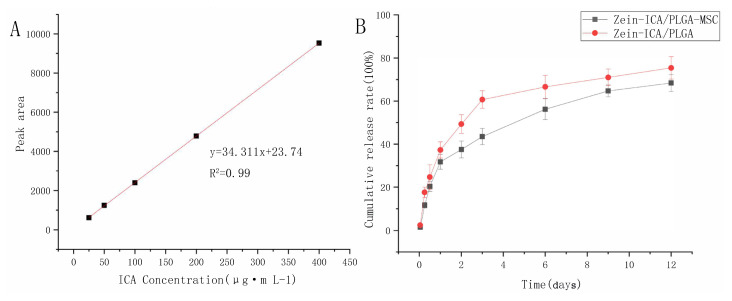
Release profile of ICA in different scaffolds.

The early and rapid release rates of ICA from Zein-ICA/ PLGA-MSC and Zein/PLGA-MSC nanofiber scaffolds (within 12 h) were 22.4% and 28.7%, respectively; from 12 h to 72 h, the release rate decreased, and the cumulative rates were 53.5% and 70.7%, respectively. After 72 h, the zein-ICA/PLGA-MSC and zein/PLGA-MSC nanofibers released 71.3% and 82.4% of the total amount of ICA, respectively. Subsequently, the release rate slowed down to a steady rate (Figure 2).

### 3.3. In vitro degradation of scaffolds

**Figure 3:**
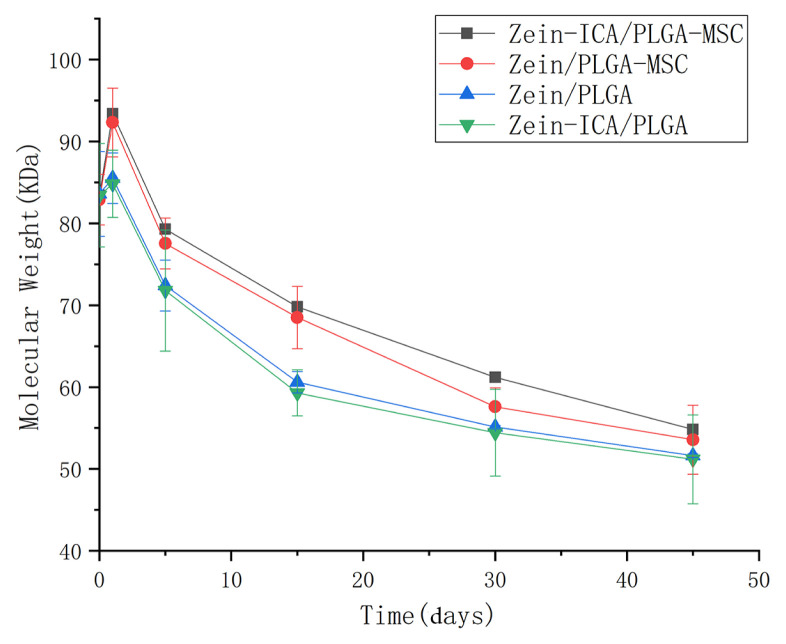
Polymer molecular weight reduction in different scaffolds.

Zein/PLGA, zein-ICA/PLGA, zein /PLGA-MSC, and zein- ICA/PLGA-MSC scaffolds were degraded in vitro within 45 days. The loading of ICA accelerated the degradation rate of the scaffolds, which was very limited. The Mw of the zein/PLGA-MSC and zein-ICA/PLGA-MSC groups decreased rapidly in the first 15 days. Meanwhile, the zein/ PLGA and zein-ICA/PLGA groups showed increased Mw reduction between days 15 and 45 (Figure 3). Although differences were observed during the degradation, the final Mw of each group was similar and did not significantly differ. Within 45 days, the Mw of zein/PLGA, zein-ICA/ PLGA, zein-ICA/PLGA and zein-ICA/PLGA-MSC decreased to 33.87%, 33.64%, 38.76%, and 38.58%.

### 3.4. Mechanical properties

**Figure 4:**
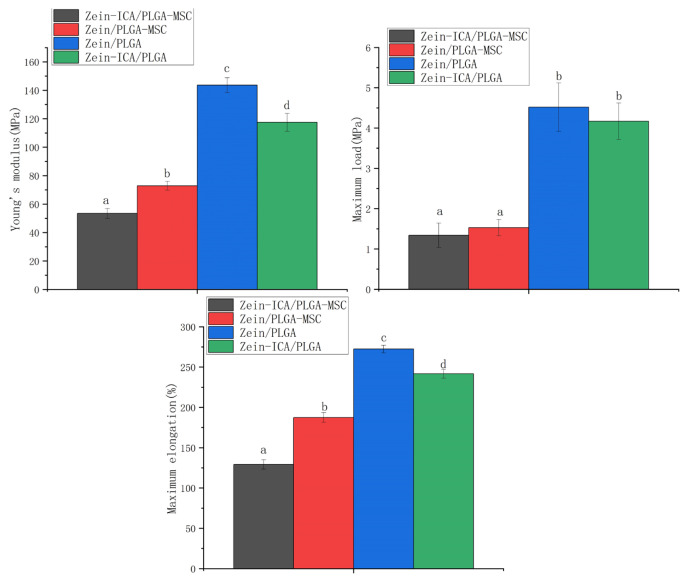
Mechanical properties of different scaffolds. a,b,c,d: Different letters represent a statistical difference.

As shown in Figure 4, the mechanical properties of the nondrug loading (zein/PLGA and zein /PLGA-MSC) groups were higher than those of the drug loading groups (zein-ICA/PLGA and zein-ICA/PLGA-MSC), and the differences in Young’s modulus and maximum elongation were significant (p = 0.0019, p = 0.0051). However, the elastic modulus of cell-loaded scaffolds was still higher than 50 MPa. The mechanical properties of the scaffolds changed considerably after the cells were combined with them, and these properties significantly decreased compared with those of noncell scaffolds (p < 0.001).

### 3.5. Cell survival rate in scaffold

**Figure 5:**
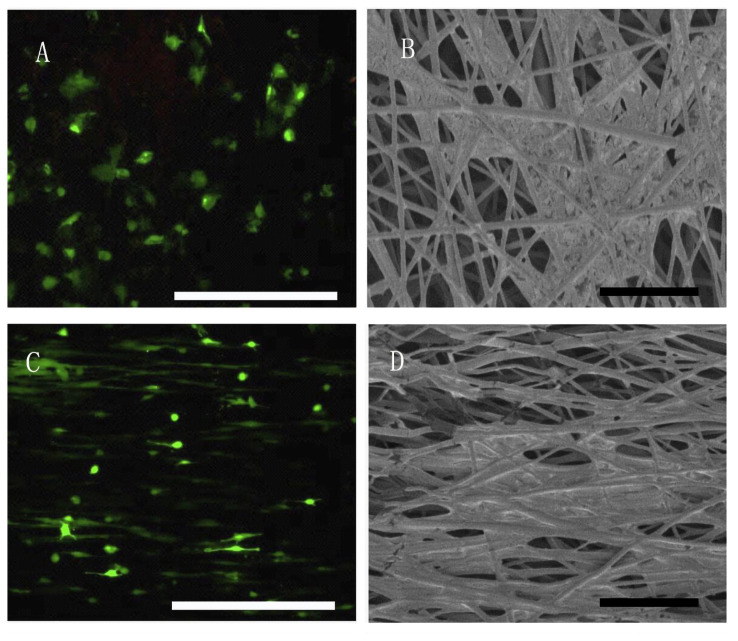
Survival rate and morphology of mesenchymal cells in nanofiber scaffolds after 7 days of incubation. (A and C) Cell activity of MSCs in zein/PLGA-MSC and zein-ICA/PLGA-MSC scaffolds, respectively; bar = 250 μm. (B and D) Morphology of MSCs in zein/PLGA-MSC and zein-ICA/PLGA-MSC groups, respectively; bar = 10 μm.

The cell scaffolds were incubated in an incubator to observe the effect of scaffold on cell viability, morphology, and differentiation. After 7 days of culture, the morphology and activity of the MSCs in the zein/PLGA-MSC and zein-ICA/PLGA-MSC scaffolds were detected via live/dead staining (Table). Figures 5A and 5C showed that the MSCs were successfully integrated into the nanofiber scaffold with uniform distribution and good cell activity. The cell morphology of the different scaffolds was analyzed via SEM. Figures 5B and 5D demonstrated that the MSCs in the zein/PLGA-MSC group had a cytoskeleton disorder. These MSCs were in fusiform shape and they had a similar fiber orientation to those in the zein-ICA/PLGA-MSC group after 7 days of incubation.

### 3.6. ALP calcium cobalt staining and ARS staining of calcified sediments

**Figure 6:**
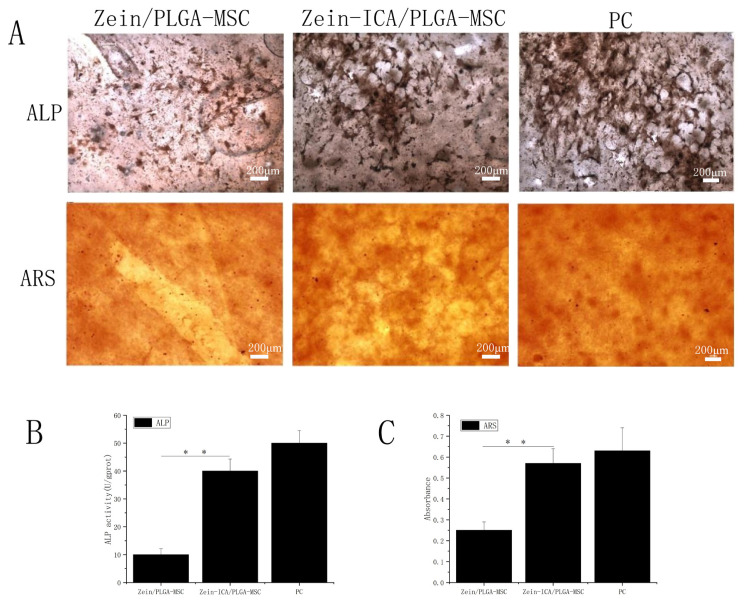
ALP staining, ARS staining, and quantitative analysis of MSCs on nanofiber scaffolds. (A) Calcium cobalt staining and ARS staining of mineralized nodules were performed 15 days after cell nanofibers were cultured in the media. (B) Quantitative analysis of ALP and ARS of the scaffolds was conducted after cell nanofibers were incubated in the medium for 15 days (*p < 0.05, **p < 0.01, # p < 0.05). Zein/PLGA-MSC incubated in osteogenic induction medium was used as positive control group.

As shown in Figure 6, the ALP expression can be observed in the zein/PLGA-MSC, zein-ICA/PLGA-MSC, and PC (zein/PLGA-MSC culture in osteogenic induction medium as positive control) fiber scaffolds after 15 days of incubation. With the passage of time, the ALP activity of all experimental groups increased. In addition, the ALP activity of cells on the zein-ICA/PLGA-MSC scaffolds was significantly higher than that on the zein/PLGA-MSC scaffolds and PC groups after 15 days of culture. This finding was related to the sustained release of ICA from the nanofibers and the maintenance of the effective concentration around the cells.

ARS staining showed that zein-ICA/PLGA-MSC and PC showed positive staining but zein/PLGA-MSC did not. Moreover, the zein-ICA/PLGA-MSC nanofiber scaffolds showed significantly dark red calcium deposits, indicating that the combination of ICA and the fiber scaffold itself accelerated the osteogenic differentiation process in the scaffolds

### 3.7. Western blot analysis of the expression of osteogenic-related proteins of MSCs in different scaffolds

**Figure 7:**
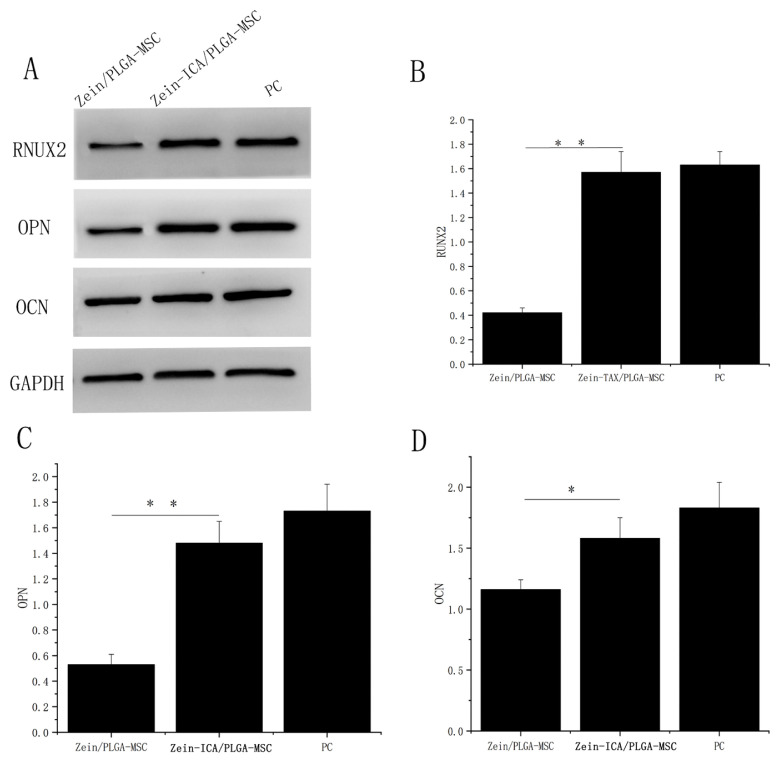
Expression of Runx2, OPN, and OCN of MSCs in different nanofiber scaffolds after 15 days of incubation. (A) Representative bands of Runx2, OPN, and OCN proteins in zein/ PLGA-MSC, zein-ICA/PLGA-MSC, and PC. (B–D) Proportional histogram of the relative expression levels of Runx2, OPN, and OCN proteins in each group (*p < 0.05, **p < 0.01, # p < 0.05). Zein/PLGA-MSC incubated in osteogenic induction medium was used as positive control group.

The expression levels of Runx2, OPN, and OCN were detected using Western blot after 15 days of osteogenic differentiation induced by nanofiber scaffolds. Figure 7 shows that Runx2, OPN, and OCN proteins were highly expressed in the zein-ICA/PLGA-MSC group. The expression of Runx2, OPN, and OCN proteins in the fibrous scaffold was significantly higher than that in the zein/PLGA-MSC group (*p = 0.0003, 0.0009, 0.0179). This result indicated that the ability of osteogenic differentiation of the scaffolds could be improved under the action of ICA drugs. Meanwhile, the expression of OPN and OCN proteins in the fibrous scaffold was significantly higher than PC group (*p = 0.0435, 0.0337). The result suggested that the ability of zein-ICA/PLGA-MSC to induce osteogenic differentiation was higher than that of osteogenic induction solution, which is consistent with previous ALP calcium cobalt staining results.

## 4. Discussion

Bone tissue engineering is a complex and rapidly developing field, which combines biomimetic scaffolds, osteoinductive, molecules and osteoblasts guiding bone regeneration to treat the severe defect (trauma, bone nonunion, etc.) or other pathological lesions (Pina et al., 2019). In bone tissue engineering of bone repair, stem cells are increasingly used to accelerate wound healing due to their good proliferation and versatility and to recover the function of damaged tissues and organs through stem cell transplantation and differentiation (Gökçinar-Yagci et al., 2015). By secreting matrix components, stem cells regulate their osteogenic differentiation and the maturation of osteoblasts and promote the formation of bone tissue, that is, the formation of a new bone (Chen et al., 2016). Bioelectrospray is a technology that places cell suspensions in electric fields and disperse them through needles to produce tiny droplets containing cells (Boda et al., 2018). Coaxial electrospinning technology overcomes the limitations of inactivation and initial burst release of bioactive molecules in traditional drug release, which is widely used in biomedicine.

In the current study, Zein/PLLA cell laden nanofiber scaffolds loaded with icariin were prepared by coaxial electrospinning and bioelectrospray for bone regeneration. Zein/PLLA nanofiber scaffolds are fabricated by electrospinning technology to simulate the extracellular matrix (ECM) for MSCs. ICA as an osteogenic inducer was loaded into nanofiber scaffolds and slowly sustained release around MSCs, forming a microenvironment of bone induction and bone conduction. SEM results show that the nanofibers are continuous and uniform in diameter, forming ECM-like structures. The fiber diameter of zein-ICA/PLGA nanofiber scaffold was larger than that of zein/PLGA nanofiber scaffold. During the preparation of Zein-ICA/PLGA nanofiber scaffolds, the conductivity of the Taylor cone surface was decreased by adding ICA into the shell solution, the tensile force of the solution was reduced, and the diameter of the fiber was increased. This could be because ICA replaced a part of zein in the Zein-ICA solution. The ICA with Mw had shorter chain length than zein, which is more likely to produce a solution with lower viscosity, resulting in the formation of fibers with considerable larger diameter and more uniform fibers (Yang et al., 2013; He et al., 2017). The diameter of zein-ICA/PLGA-MSC was larger than that of zein-ICA/PLGA; this may be due to the fact that continuous contact with the medium from the cell suspension may result in fiber expansion and subsequent increase in diameter.

Coaxial electrospinning is the main development of electrospinning technology. It can form two concentric layers with different characteristics, isolate various stimulating factors in different fiber material layers, and adjust the release characteristics by changing the thickness and position of fiber that overcomes the limitations of traditional delivery system, such as inactivation and sudden release of bioactive molecules (Shalumon et al., 2015). ICA did not show a high initial burst release in both cell-loaded and noncell-loaded scaffolds, and it entered a slow release phase after 12 h. This is different from single-layer fiber. It is widely known that the drug loaded by single-layer fiber can be exposed to the surrounding environment on the fiber surface, and most of them show relatively high initial burst release (Alhusein et al., 2013). The mechanism of drug release is diffusion and polymer degradation (Casalini et al., 2014; Zhu et al., 2015). The fluid around the material penetrates into the inner layer of the polymer through the scaffold structure and dissolves the drug to make it diffuse or filter the drug out of the polymer matrix into the surrounding environment. A release profile of ICA showed that the release mechanism of ICA may be initially through the pore diffusion in the fibrous scaffold structure and later became a diffusion/ erosion coupling mechanism (Abdullah et al., 2019; Lima et al., 2019).

The addition of cells decreased the Young’s modulus load and maximum elongation of zein/PLGA, because the substitution of ICA for zein reduced the entanglement of long chains, and the cells interrupted the integrity of the fiber network, thus resulting in a decrease in the mechanical strength of scaffolds. It could also be attributed to the viscosity of the solution increases after PLGA solution in the core layer is loaded with cells. Under static electricity, the dispersion of the solution becomes more difficult and the stability of electrospinning was also affected, resulting in increasing of fiber diameter from 1.3 ± 0.12 μm to 1.835 ± 0.16 μm. The broadened distribution range of fiber diameter leads to uneven stress on the fiber and affects its mechanical properties.

Scaffolds should provide a framework for cell development until tissue regeneration, and the stability of scaffolds is very important for the maintenance of reasonable space for cell proliferation. The degradation of scaffolds is closely related to the stability of scaffolds in vivo (Do et al., 2015; Singh et al., 2020). In the present study, the degradation experiments showed that zein/PLGA, zein-ICA/PLGA, zein /PLGA-MSC, and zein-ICA/PLGA-MSC had different degradation kinetics. In the first 15 days, the decrease in the Mw of scaffolds may indicate degradation of its surface. Besides, the decrease in Mw between days 15 and 45 may represent internal degradation of the scaffolds. Although the degradation kinetics was different, no significant difference was found in the final Mw. Therefore, the combination of bioelectrospray and coaxial electrospinning did not cause any significant difference in fiber degradation rate. Although cell integration affected some physicochemical properties of scaffolds, their properties could still meet the needs of tissue engineering applications.

Superfine fiber scaffold is a matrix, on which surface is conducive to cell adhesion and diffusion (Lu et al., 2014; Honarvar et al., 2022; Sattary et al., 2022). Rational space structure of scaffold in line with good nutrient transport and metabolism exchange is closely related to the improvement of cellularity (Bao et al., 2019). The results of live/dead staining and the fluorescence images showed that the MSCs were successfully integrated into the nanofiber scaffold and distributed evenly with good cell viability and had directional arrangement during the culture. The SEM image demonstrated that the cells existed above and below the fiber network. The physical structure of zein-ICA/PLGA-MSC provided a suitable environment for the development of embedded MSC, MSC in zein-ICA/PLGA-MSC grew well and were arranged orderly, which is consistent with the results of fluorescence images. Although the scaffolds prepared by electrospinning have good compatibility with stem cells, most of them are used very limitedly to realize uniform distribution and directional growth of stem cells (Deliormanlı et al., 2018; Sattary et al., 2022). Since the introduction of bioelectrospraying seeding method, the cell laden scaffold material has uniform cell arrangement and directional growth. In addition, the arrangement of trabeculae in cancellous bone has directionality and relative homogeneity. Well-arranged trabeculae parallel to the direction of stress is conducive for bone tissue to bear higher stress (Akhtar, 2007). The directional arrangement of uniform distribution of MSCs in the prepared scaffold is of great significance to develop normal bone structure (Matsugaki et al., 2020).

Zein/PLGA-MSC nanofiber scaffolds loaded with ICA showed good osteogenic differentiation ability. The bioactive molecule ICA was successfully loaded into the newly prepared Zein/PLGA-MSC coaxial electrospinning nanofiber scaffold and maintained its bioactivity. The drug was effectively released around the MSC in the scaffolds and induced them to differentiate into osteoblasts. This finding is consistent with studies using silk fibroin as scaffolds incorporated with vitamin D3(Mostafavi et al., 2022). During the differentiation process, Runx2, OPN, and OCN proteins related to osteogenesis were highly expressed, which indicated that Zein/PLGA nanofiber scaffold loaded with ICA could achieve osteogenic differentiation and bone regeneration of MSCs through the Runx2, OPN, and OCN pathways.

## 5. Conclusion

The new nanofiber scaffolds showed good biocompatibility. MSC could survive and proliferate in the scaffold. The MSCs in the zein-ICA/PLGA-MSC scaffolds showed high proliferation rate and directional arrangement. The slowly sustained release of ICA in the nanofiber scaffolds promoted the cells in the nanofiber scaffolds to show good cell biological activities. The MSC in the zein-ICA/ PLGA-MSC scaffolds showed higher ALP activity and ECM mineral deposition than those in the zein/PLGA-MSC scaffolds. The slow release of ICA and the uniform distribution of MSC nanofiber scaffold are expected to provide rapid and effective bone induction suitable for bone tissue engineering applications.

## Figures and Tables

**Table:** Live and dead cells quantification.

Group	Living cells	Dead cells	Survival rate (%)	Cell mortality (%)
Zein/PLGA-MSC	131 ± 12	46 ± 8	74.01	25.99
Zein-ICA/PLGA-MSC	152 ± 14	58 ± 17	69.09	27.61
